# What leads to parallel evolution?

**DOI:** 10.7554/eLife.73553

**Published:** 2021-10-18

**Authors:** Debapriyo Chakraborty

**Affiliations:** 1 Host Pathogen Interaction Unit (IHAP), École Nationale Veterinaire de Toulouse Toulouse France; 2 Institut National de la Recherche pour l’Agriculture , l’Alimentation et l’Environnement (INRAE) Toulouse France

**Keywords:** influenza virus, evolution, RNA interactions, Virus

## Abstract

The repeated emergence of similar variants of influenza virus is linked to interactions between the virus’s RNA segments.

**Related research article** Jones JE, Le Sage V, Padovani GH, Calderon M, Wright ES, Lakdawala SS. 2021. Parallel evolution between genomic segments of seasonal human influenza viruses. *eLife* **10**:e66525. doi: 10.7554/eLife.66525

Scientists around the world are busy predicting how SARS-CoV-2 – the virus responsible for the COVID-19 pandemic – will spread so it can be tracked and stopped from advancing further (Vespignani et al., 2020). Similarly, if we were able to predict how viruses evolve, that could help us track and prevent pandemics in the future. Unfortunately, predicting virus evolution is extremely difficult (Geoghegan and Holmes, 2017), but evolutionary biologists have noticed that some viral variants appear repeatedly, following similar changes in their environment. Fully understanding this phenomenon – which is called parallel evolution (Gutierrez et al., 2019) – would be an important step towards being able to predict how viruses evolve and, ultimately, being able to forecast pandemics.

Now, in eLife, Seema Lakdawala, Erik Wright and colleagues at the University of Pittsburgh – including Jennifer Jones as first author – report the results of a study that investigated parallel evolution in human influenza viruses (Jones et al., 2021). Specifically, they studied viral genomic reassortment, which is a major driver of influenza evolution (Figure 1A). Genomic reassortment usually occurs in viruses that have a genome that is divided among two or more nucleic acid molecules. The genome of the influenza virus, for example, is divided among eight different RNA segments (namely PB2, PB1, PA, HA, NP, NA, M and NS).

**Figure 1.**
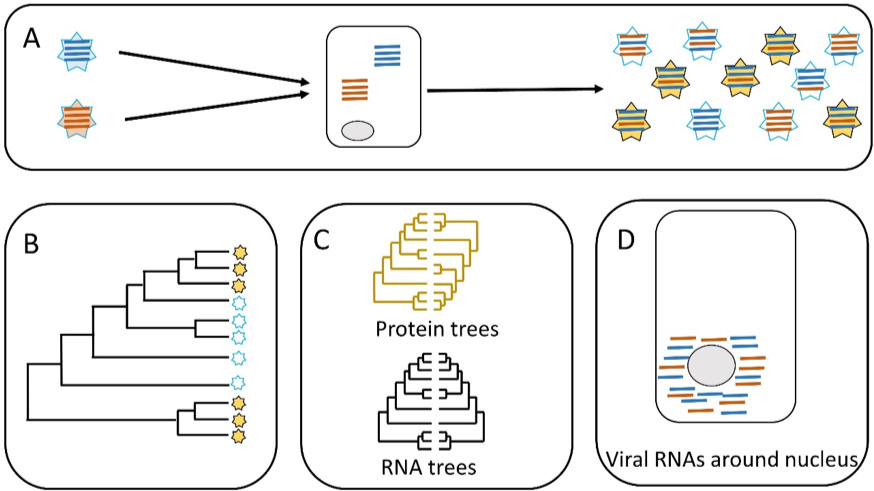
Parallel evolution in influenza viruses. (**A**) Two virions with different genomes (left) infect a cell, and the RNA segments in the virions (blue and red lines) are released into the cytoplasm. The RNA segments replicate, and the new segments are then assembled into new virions (right). Some of the new virions are similar to the original virions (all blue or all red segments) but many are different (i.e. both blue and red segments). (**B**) Sometimes similar progeny variants emerge repeatedly due to parallel evolution. (**C**) Jones et al. showed that the phylogenetic trees for some of the RNA segments of influenza A are similar (black), whereas the phylogenetic trees for the proteins that these RNA segments encode are different (yellow). (**D**) Microscopy demonstrates that RNA segments localize around the nucleus of the host cell during infection, permitting direct interaction between the segments. These two lines of evidence (phylogenetics and microscopy) point to RNA–RNA interactions having a role in the parallel evolution of viruses.

When an influenza virion infects a host cell, it releases these RNA segments into the cytoplasm, where copies are made by the replication machinery of the host. These copies are then assembled into new virions. Importantly, a new virion can, in theory, contain copies of segments from any of the virions that originally infected the cell. This process of reassortment helps to maintain the high level of genetic diversity that the virus requires for rapid adaptation and evolution. Remarkably, reassortment between just two influenza virions can create up to 254 new combinations of segments (Lowen, 2017).

After reassortment, each new virion needs to have eight segments of the right type to be viable (that is, one PB2 segment, one PB1 segment, and so on). This means that reassortment cannot be a random event where any segment can combine with any other segment. Instead, segments need to be arranged in some ordered fashion to avoid the assembly of virions that have two (or more) copies of the same type of segment. This process is likely driven by interactions between the RNA segments, although the exact nature of such interactions is unclear (Lowen, 2017).

It has been hypothesized that disrupting the interactions between a pair of RNA segments could hinder the interactions between other segments (Dadonaite et al., 2019). Therefore, it follows that these RNA-RNA interactions are an important constraint on reassortment and, in turn, on the parallel evolution of influenza viruses. To test this hypothesis, Jones et al. collected genomic sequences for two strains of influenza (namely H1N1 and H3N2) from online databases, and used these sequences to construct phylogenetic trees for each of the eight types of segments.

Jones et al. predicted that the phylogenetic trees of different segments would be similar across time when similar viruses emerge repeatedly: in other words, the presence of similar phylogenetic trees in different segments would be evidence for parallel evolution (panels B and C in Figure 1). The phylogenetic trees built by Jones et al. supported parallel evolution in human influenza viruses, but the level of parallel evolution varied between the H1N1 and the H3N2 strains, and also between variants of the H1N1 strain. For instance, the study found strong evidence for the parallel evolution of segments PB1 and PA, which both have roles in RNA replication; however, the evidence for the parallel evolution of PB1 and HA (which have different roles in cells) was weak. This connection between parallel evolution and the role of the different segments paints a picture of diverse interactions between the different strains of the virus and their environment.

The second hypothesis tested by Jones et al. distinguished between two scenarios. In the first scenario, the RNA segments interact directly with each other when they are free in the cell. In the second scenario, the RNA segments interact indirectly via their proteins (Figure 1C). Evidence to support the role of protein-protein interactions in the parallel evolution of viruses (i.e. the second scenario) has already been reported, so Jones et al. wanted to investigate the role of RNA-RNA interactions. To do this, they built phylogenetic trees for the viral proteins and compared them. This helped them to identify proteins that do not show evidence of parallel evolution. Now they focused on RNA segments that encoded these proteins. They found that some of those RNA segments had similar phylogenetic trees. This is clear evidence of a direct role for RNA-RNA interactions in the parallel evolution of influenza. Finally, Jones et al. used molecular techniques and microscopy to show that the RNA segments co-localize within the cell, which needs to happen for RNA-RNA interactions to occur (Figure 1D).

The detection of parallel evolution in viruses can aid surveillance by identifying repeated patterns of viral emergence, while understanding the mechanisms that underpin parallel evolution may help researchers to predict the emergence of specific strains. The work of Jones et al. represents considerable progress towards both goals. It also establishes phylogenetic analysis as a useful tool for investigating basic questions related to viral evolution, and applied questions related to epidemic surveillance. However, to fully understand parallel evolution we still need a more complete understanding of the links between genomic mechanisms involved, and emergent phenotypes, the environment and host-virus interactions.
